# The effect of risk factors on cognition in adult cochlear implant candidates with severe to profound hearing loss

**DOI:** 10.3389/fpsyg.2022.837366

**Published:** 2022-08-16

**Authors:** Miryam Calvino, Isabel Sánchez-Cuadrado, Javier Gavilán, Luis Lassaletta

**Affiliations:** ^1^Department of Otolaryngology, Hospital Universitario La Paz, IdiPAZ Research Institute, Madrid, Spain; ^2^Biomedical Research Networking Centre on Rare Diseases (CIBERER), Institute of Health Carlos III, Madrid, Spain

**Keywords:** severe to profound hearing loss, cognition, risk factors, education, age, habits, RBANS-H

## Abstract

Hearing loss has been identified as a major modifiable risk factors for dementia. Adult candidates for cochlear implantation (CI) represent a population at risk of hearing loss-associated cognitive decline. This study investigated the effect of demographics, habits, and medical and psychological risk factors on cognition within such a cohort. Data from 34 consecutive adults with post-lingual deafness scheduled for CI were analyzed. Pure tone audiometry (PTA4) and Speech Discrimination Score (SDS) were recorded. The Repeatable Battery for Assessment of Neuropsychological Status for Hearing impaired individuals (RBANS-H) was used to measure cognition. Demographics (sex, age, years of education), habits (smoking, alcohol intake, physical inactivity), and medical factors (hypertension, diabetes, traumatic brain injury) were evaluated. Depression was measured using the Hospital Anxiety and Depression Scale (HADS), and social inhibition with the Type D questionnaire (DS14). All participants (mean age 62 ± 15 years) suffered from severe to profound hearing loss (PTA4:129 ± 60 dB; SDS:14 ± 24%). The mean RBANS-H total score was 83 ± 16. Participants reported a mean of years of formal education of 12 ± 5 years. The prevalence of habits and medical risk factors was: physical inactivity (29%), body mass index >30 (28%), traumatic brain injury (25%), hypertension (24%), heavy alcohol consumption (13%), smoking (13%), and diabetes (0%). Regarding psychological factors, the mean scores of social inhibition and depression were 10 ± 6 and 6 ± 5, respectively. The number of years of education was significantly correlated with the RBANS-H total score (*p* < 0.001), and with the domains “Immediate memory” (*p* = 0.003), “Visuospatial/constructional” (*p* < 0.001), and “Attention” (*p* < 0.001). The mean RBANS-H total score in participants who had university studies or higher level (12/34) was 97 ± 9, with the remaining participants reporting a mean score of 75 ± 15. Men performed better in the “Visuospatial/constructional” (*p* = 0.008). Physical inactivity was associated with lower scores in the “Delayed memory” (*p* = 0.031); hypertension correlated with lower RBANS-H total scores (*p* = 0.025) and “Attention” (*p* = 0.006). Depression and social inhibition were negatively correlated with RBANS-H total score and with the “Immediate memory,” “Visuospatial/constructional,” and “Attention” (all *p* < 0.05). In adults with late-onset deafness scheduled to CI, educational level has a significant effect. Additionally, sex, physical inactivity, hypertension, and psychological traits of social inhibition and depression may also influence cognitive status. Long-term studies with more participants would enable us better understand the effects different risk factors on cognitive status.

## Introduction

Dementia affects an estimated 55 million people worldwide. Due to aging demographics and the reduction in younger age mortality, this figure is expected to rise to over 139 million by 2050 (WHO, 2021a). Dementia influences practically all aspects of life and as such represents a major psychological, social, and medical burden.

Alzheimer’s disease and other forms of dementia are among the 10 leading causes of death worldwide, and were the third highest cause of mortality in upper income countries in 2019 (WHO, 2020). Due to age, multimorbidity, and difficulties in maintaining social distance, people with dementia are at especially high risk of COVID-19-related illness (Wang et al., 2020). Some authors regard dementia as the greatest universal challenge for social and health care in the 21st century (Chern and Golub, 2019).

**Figure 1:**
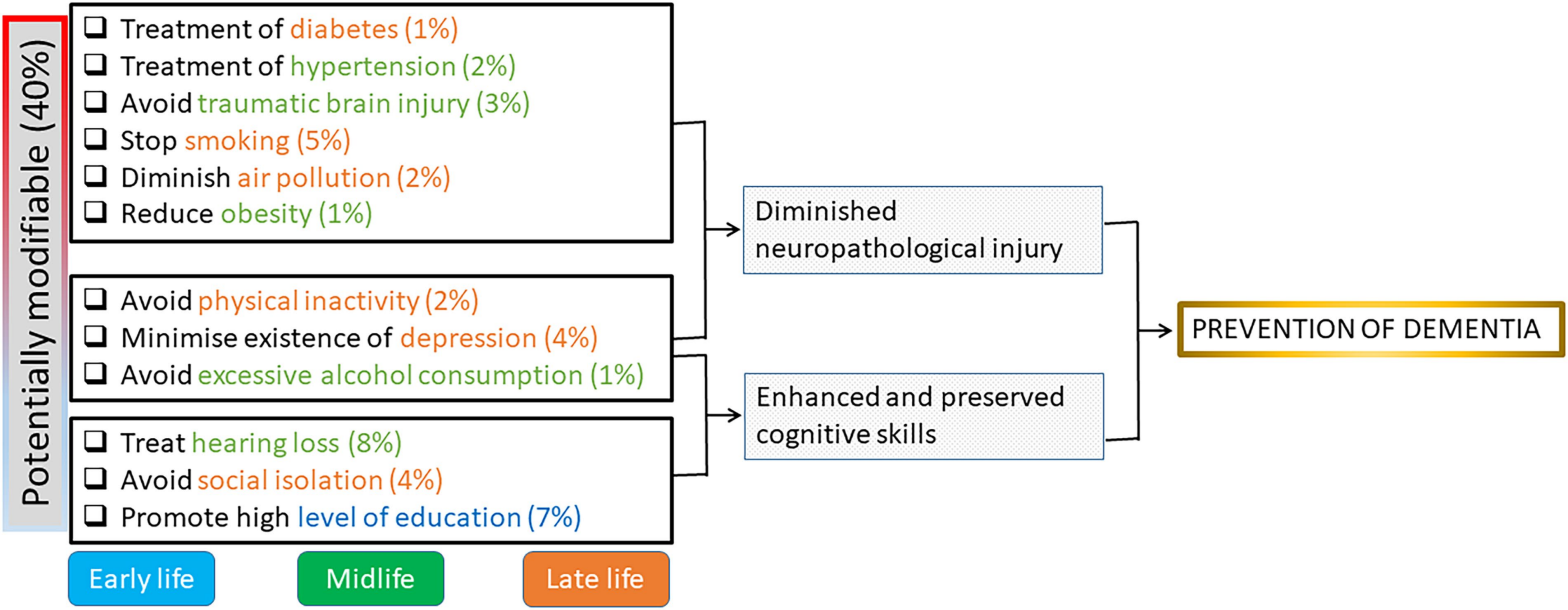
Potential tools of protection (modifiable risk factors) for preventive actions in cognitive decline and the estimated percent of prevented cases. Early life is <45 years; Midlife is 45–65 years, Late life is >65 years. Modified from Livingston et al. (2020).

The 2017 Lancet commission model identified nine modifiable risk factors for dementia: less education, hypertension, hearing impairment, smoking, obesity, depression, physical inactivity, diabetes, and low social contact (Livingston et al., 2017). In 2020, three additional modifiable risk factors were identified: excessive alcohol consumption, traumatic brain injury, and exposure to air pollution (Livingston et al., 2020). The authors calculated that 40% of cases of dementia could be prevented by modifying these 12 risk factors (Figure 1). As Livingston et al. stated “it is never too early and never too late in the life course for dementia prevention” (Livingston et al., 2020).

Aside from those found in the Lancet commission reports, other studies have found additional risk factors. Recently, a prospective study was conducted with 727 Korean participants (≥65 years old) with no cognitive disorder at recruitment concluded. This study identified several risk factors for age-related cognitive decline, including low socio-economic status (poor education, low income, living in a rural area), unhealthy behaviors (unintentional weight loss, reduced hand grip strength), and health conditions (depression; Kang et al., 2021). Likewise, demographic features such as sex and marital status have been identified as risk factors for age-related dementia (Legdeur et al., 2018; Zhang et al., 2019). There are strong indications that healthy behaviors like good nutrition (Smith and Blumenthal, 2016; Zhang et al., 2019) and physical fitness (Daimiel et al., 2020) are associated with improved cognition.

Given the fact that drug treatment options for dementia are currently limited in terms of efficacy (Yu et al., 2021), it is essential to focus on modifiable risk factors to delay or prevent dementia. This is especially true in light of the fact that dementia is easier to prevent than to treat once established (Lin et al., 2011). Hearing loss has been identified as one of the most prominent modifiable risk factors for cognitive decline (Livingston et al., 2017). It is estimated that by 2050 nearly 2.5 billion people (1 in 4) will have some degree of hearing impairment, and at least 700 million (7% of the world’s population) will need hearing rehabilitation (hearing aids or a hearing implant; WHO, 2021c). This fact has not yet been adequately prioritized in the treatment strategy for individuals with cognitive disorders, or those who are at risk of developing them.

So, the aim of this study was to investigate the effect upon cognition of education level, hypertension, smoking, obesity, depression, physical inactivity, diabetes, reduced social contact, heavy alcohol consumption, and traumatic brain injury in late-deafened adults who were candidates for cochlear implantation.

## Materials and methods

### Design

This cross-sectional study was performed at the Department of Otorhinolaryngology, La Paz University Hospital, Madrid, Spain. The research took place from October 2016 to November 2020. Measurements of cognition, audiological evaluation, and questionnaires about risk factors were carried out just prior to cochlear implantation. The project was approved by the local Ethics Committee (protocol number: PI-2504). Participants gave their written informed consent prior to study procedures.

### Participants

Participants were recruited among consecutive adults with bilateral severe to profound sensorineural hearing loss who were candidates for cochlear implant (CI) provision at the Cochlear Implant Unit of La Paz University Hospital, Madrid, Spain. Criteria for cochlear implantation were adults (≥18 years) with a mean post-lingual hearing loss of >71 dB at 500, 1,000, 2,000, and 4,000 Hz (i.e., PTA4) who derive insufficient benefit from hearing aid use (<40% of disyllabic words at 65 dB; Lassaletta et al., 2019). Moreover, they should not have any neurological, cognitive, or severe visual impairment in their medical history in order to be able to complete the specific tests of this study.

### Measures

#### Cognitive evaluation

**Figure 2:**
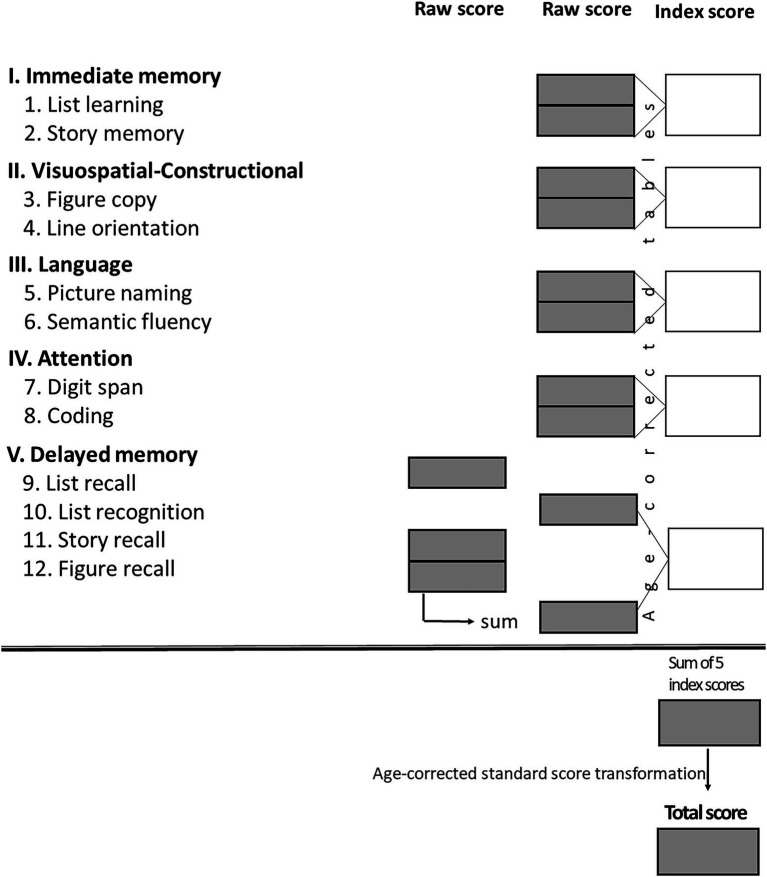
Score conversion sheet with the five domains (I–V) and the twelve subtests (1–12). The sum of the 5 index scores results in a final RBANS-H score which is transformed into an age-corrected standardized score (“total score”) with a mean of 100 and a standard deviation (SD) of 15 points – average cognitive status is considered 100 points according to a Gaussian distribution, score which equates to the 50th percentile (Claes et al., 2016).

The Repeatable Battery for the Assessment of Neuropsychological Status for Hearing impaired individuals (RBANS-H; Claes et al., 2016) was used to assess cognition in individuals with hearing impairments. This is a modification of the RBANS battery (Randolph et al., 1998) in which oral instructions are supplemented by written instructions delivered on presentation slides to reduce the influence of hearing impairment on test performance. The scoring sheet for RBANS-H is shown in Figure 2. Generally, the raw scores for each domain subtest are compiled into an index score for that domain, which are then transformed into an age-corrected total score for overall performance.

#### Audiological evaluation

Audiological evaluations were performed using a two-channel Madsen Astera^2^ audiometer (Otometrics, Taastrup, Denmark) in an acoustically treated room. In cases where the participant had better hearing in their contralateral ear, it was masked during assessment. PTA4 and speech recognition ability (SDS, maximum Speech Discrimination Score) in both ears were evaluated.

#### Assessment of cognition-associated risk factors

##### Socio-demographics

- Age and sex. Obtained from medical records.
- Education level. Years of formal education were determined from an interview questionnaire. Categories were determined by the total number of years spent in formal education since the age of 6 years: primary education (8 years), secondary education (12 years), university studies, master’s degree or PhD (≥17 years).

##### Habits

Habits were as per interview questionnaire responses.

- Smoking: (yes/no).
- Heavy alcohol consumption: (yes/no). “Heavy” was defined as 5 or more alcoholic drinks per week (Plunk et al., 2014).
- Physical inactivity: (yes/no). Level of activity was quantified as number of minutes spent on physical activity during a normal week: physically inactive was <150 min per week; physically active: >150 min per week (Gerst et al., 2011).

##### Medical factors

Medical factors were also as per interview questionnaire, and later corroborated by medical records:

- High blood pressure: (yes/no). Participants were asked if they had a medical diagnosis of hypertension, regardless of if they were under treatment.
- Medical diagnosis of diabetes: (yes/no). Participants were asked if they had been diagnosed with type 1 or type 2 diabetes.
- Traumatic brain injury: (yes/no). Participants were asked if they had had either a concussion or skull fracture, edema, brain injury, or brain bleeding at some point in their life (Livingston et al., 2020).
- Obesity: (yes/no). BMI ≥ 30 kg/m^2^, as per WHO guidelines, was evaluated from measurements of height and weight (WHO, 2021b).

##### Psychological factors

Social inhibition was evaluated by means of Type D questionnaire (DS14).The DS14 questionnaire, validated in the Spanish population (Alcaraz et al., 2018), is broadly used to evaluate the existence of type D (distressed) personality, which is characterized by the traits of negative affectivity and social inhibition (Denollet, 2005). To assess the social inhibition component, the questionnaire comprises seven items (e.g., “I would rather keep other people at a distance”) that are scored from 0 to 4 (False to True). A score of ≥10 indicates high social inhibition (Timmermans et al., 2019).

Depression was evaluated *via* the Hospital Anxiety and Depression Scale (HADS) questionnaire. The subscale of HADS that evaluates depression is comprised of seven items (e.g., “I still enjoy the things I used to enjoy”). Participants rate each subscale from 0 to 3 points, scores are added to obtain a range of 0–21 points. Higher scores indicate depressive manifestations (Zigmond and Snaith, 1983). According to Snaith (2003), a score of 0–7 indicates a normal range, 8–10 is a borderline case, and 11 or higher indicates a high probability of depression (“caseness”).

### Data analysis

Demographic characteristics and outcome measures are presented as absolute values, percentages and, where appropriate, the mean and ±SD are provided.

Pearson’s correlation coefficient was calculated to evaluate the relationship between cognition (RBANS-H), demographic data (age and years of formal education), audiometric data (PTA4 and SDS), and the corresponding subscales of DS14 and HADS questionnaire scores.

Analysis of variance (ANOVA) was used to measure the association between cognition and sex, smoking, alcohol intake, physical activity, hypertension, diabetes, traumatic brain injury, and obesity.

Missing data and the response option “Not applicable” were treated as missing values. A level of *p* ≤ 0.05 (2-tailed) was considered significant. Statistical analysis was performed with the SPSS software package v24.0 (IBM Corp., Armonk, NY, United States).

## Results

### Participant demographics

Table 1 summarizes the participant demographics. Sex, age, years of formal education, hearing loss duration, and the use of a hearing aid in the contralateral ear are recorded.

**Table 1:** Demographic data.

Demographic variable		*n*
Sex	Female (*n*) (%)	19/34 (56%)
	Male (*n*) (%)	15/34 (41%)
Age (years) (mean ± SD)		62 ± 15
Years of formal education (mean ± SD)		12 ± 5
None (*n*) (%)		3 (9%)
Primary education (*n*) (%)		12 (35%)
Secondary education (*n*) (%)		7 (21%)
University studies, master’s degree or PhD (*n*) (%)		12 (35%)
Hearing loss duration (years) (mean ± SD)		28 ± 16
Use of hearing aid in the contralateral ear (*n*) (%)		21 (62%)

SD, standard deviation.

### Cognitive status

**Figure 3:**
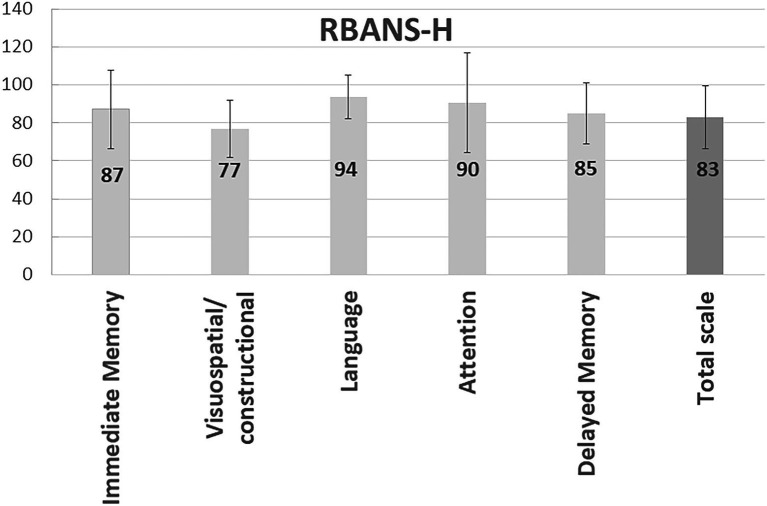
Measurement of cognition in study participants. The bars represent the mean and standard deviation of the RBANS-H total and domains scores.

The mean scores in the total RBANS-H score and domains are shown in Figure 3. The RBANS-H assessment is standardized, with 100 points (the 50th percentile) being considered as average in cognitive abilities. In all domains, mean scores for participants were lower than the population, with the highest scores achieved in the “Language” domain (94 ± 11), and the lowest scores reported in the “Visuospatial/constructional” domain (77 ± 15). For all combined subscales, i.e., RBANS-H total score, the participants were found to have a mean score of 83 ± 16 (22th percentile).

### Cognition-associated risk factors

#### Audiometric data and speech recognition outcomes

Table 2 summarizes the audiometric outcomes in terms of PTA4 and percentage of correctly identified disyllabic words in silence (speech discrimination score, SDS). Results of both ears are shown.

**Table 2:** Audiometric outcomes in both ears evaluated separately.

	Mean ± SD
PTA4 (dB)^*^	
Ear to be implanted	129 ± 60
Contralateral ear	95 ± 27
SDS maximum (%)	
Ear to be implanted	14 ± 24
Contralateral ear	47 ± 34

PTA4: mean pure-tone audiometry values at 500, 1,000, 2,000, and 4,000 Hz; SDS: speech discrimination score. ^*^A value of 140 dB was used if no response was detected.

#### Prevalence of habits, medical and psychological factors

**Figure 4:**
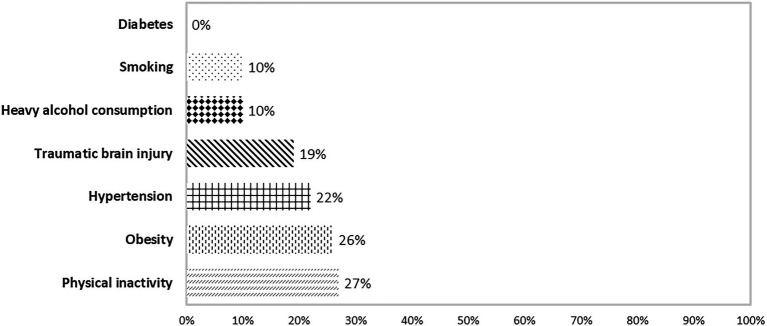
Prevalence of cognition-associated risk factors related to habits and medical and psychological condition reported by participants.

The prevalence of the self-reported habits and medical factors were: diabetes (0%), smoking (10%), heavy alcohol consumption (10%), traumatic brain injury (19%), hypertension (22%), obesity (26%), and physical inactivity (27%; Figure 4).

Regarding psychological factors, participants showed elevated social inhibition on the DS14 scale, with a mean value of social inhibition of 10 ± 6 (with a score of ≥10 indicating high social inhibition). The mean score for depression, as evaluated by HADS, was 6 ± 5, indicating a low probability of depression in this cohort (0–7 indicates a normal range).

### Effect of risk factors on cognitive function

#### Demographic factors

*Age*: no significant association was found between the age of our participants and their cognitive skills.

*Sex*: men outperformed women in the “Visuospatial/constructional” domain (*p* = 0.008; means 84 ± 14 vs. 71 ± 13).

*Education:* Participants who had attended university studies had a higher mean RBANS-H total score than participants who did not attend university (97 ± 9 vs. 75 ± 15). The number of years of education was significantly correlated with the RBANS-H total score (*r* = 0.653, *p* < 0.001), and with the domains “Immediate memory” (*r* = 0.493, *p* = 0.003), “Visuospatial/constructional,” (*r* = 0.658, *p* < 0.001) and “Attention” (*r* = 0.647, *p* < 0.001).

#### Hearing level

Speech Discrimination Score values in the ear to be implanted showed a trend of correlation with the domain “Delayed memory” (*r* = 0.345, *p* = 0.050).

#### Habits

Physically inactive participants had lower scores in the “Delayed memory” domain (77 ± 22 vs. 91 ± 9, *p* = 0.031). Moreover, a trend of correlation was observed with the domains of “Attention” (78 ± 32 vs. 101 ± 22, *p* = 0.066), and the RBANS-H total score (77 ± 20 vs. 90 ± 14, *p* = 0.095). Neither smoking nor heavy alcohol consumption had a significant effect on any RBANS-H cognitive domain.

#### Medical factors

The presence of a diagnosis of hypertension was associated with worse scores in the “Attention” domain (57 ± 24 vs. 103 ± 23, *p* = 0.006) and in the RBANS-H total score (57 ± 18 vs. 91 ± 14, *p* = 0.025). Neither the presence of obesity nor of traumatic brain injury were associated with worsened outcomes in any RBANS-H domain.

#### Psychological factors

RBANS-H total score was negatively correlated with the depression and social inhibition subscales; moreover, most of the RBANS-H domains were also inversely correlated with both psychological factors (see Table 3).

**Table 3:** Correlations between the RBANS-H and the psychological factors depression and social inhibition.

		Depression	Social inhibition
RBANS-H	Immediate memory	−0.380	−0.595
		0.027	<0.001
	Visuospatial/constructional	−0.595	−0.388
		<0.001	0.024
	Language	NS	NS
	Attention	−0.584	−0.469
		<0.001	0.005
	Delayed memory	NS	NS
	Total score	−0.566	−0.517
		<0.001	0.002

Top – Pearson’s coefficient. Bottom – significance level (*p* < 0.05). NS, not significant.

## Discussion

In this cross-sectional study, we analyzed the effect of demographics, habits, medical and psychological risk factors on the cognitive status of adults with late post-lingual deafness who were CI candidates. Cognitive status was measured with a specific tool for people with hearing impairment, RBANS-H. Our results revealed that a higher cognitive status was correlated with higher educational attainment. Participants who were male, did not suffer from hypertension, or did regular physical activity had better results in specific domains related to cognition. Scores in both of the psychological risk factors, depression and social inhibition, inversely correlated with the RBANS-H scores.

### Cognitive abilities and hearing impairment

It has been shown that the risk of cognitive decline is higher in individuals with hearing impairment than in those with normal hearing (Uhlmann et al., 1989; Claes et al., 2018). In fact, hearing loss has been considered the modifiable risk factor with the highest potential for dementia mitigation (weighted population attribution fraction of 8%; Livingston et al., 2020). Generally speaking, patients with hearing loss begin to seek treatment about 7 years after the onset of symptoms (NIDCD, 2018). This delay can mean that, by the time treatment is initiated, hearing loss may have progressed beyond the point at which irreversible effects upon cognition have occurred.

Different theories have been suggested to explain the connection between hearing impairment and cognition (Wayne and Johnsrude, 2015; Uchida et al., 2019; Amieva and Ouvrard, 2020):

- Cognitive load hypothesis: the cognitive effort to concentration and attention that an individual needs to carry out in a task is known as “cognitive load.” If the subject suffers from hearing loss, listening effort during speech perception is always present; it is similar to perform “dual tasks” at the same time, and if there is a high effort for the most important task, a decrease in the performance of the secondary task will be observed. When there is hearing difficulty, the neural activity demanded for speech understanding is higher if the brain has to mobilize extra neural populations for a good performance (Lindenberger and Baltes, 1994).
- Common cause hypothesis: according to this theory, both hearing impairment and cognitive decline may result from a common neurodegenerative factor in the brain (Lindenberger and Baltes, 1994). Both processes are multifactorial and risk factors coexist and might vary, p.e. oxidative stress, microcirculatory insufficiency, genetics (APOE gene), and physical health (Wayne and Johnsrude, 2015).
- Cascade or information-degradation hypothesis: according to this hypothesis, cognitive decline is because the decrease in hearing levels (a poor sensory information) leads to an alteration of brain structure. Social isolation, physical inactivity, depression, and other detriments related to hearing loss due to a decrease in speech perception and communication, could accelerate cognitive decline according to the fact that processing abilities could be lost if they are no used any more (Uchida et al., 2019).
- Overdiagnosis or sensory deprivation hypothesis: one of the potential reasons for the relationship between hearing impairment and cognitive function is overdiagnosis. Here, the performance on specified neuropsychological tests, is influenced by a sensory deprivation (hearing), rather than cognition. So, it is critical that a cognitive test intended to be used in populations with severe hearing impairment be designed so that cognition scores are independent of hearing ability, in order not to waste cognitive resources on the task of orally understanding the test which leads an overestimation of the level of cognitive decline (Lin et al., 2013). In this study, the RBANS-H neuropsychological test battery was used for cognition evaluation. This is a modification of the RBANS test in which oral instructions are supplemented by written instructions delivered on presentation slides, to reduce the effect of hearing impairment on test performance (Claes et al., 2016).

Our study showed a trend in which poorer speech discrimination scores were associated with worse scores in the “Delayed memory” domain of the RBANS-H. This finding is in accordance with previous reports about the association between hearing loss and cognition (Livingston et al., 2020). However, it should be kept in mind that all participants had severe to profound deafness, so there was a small variation in terms of speech discrimination.

It has been suggested that mental substitution or phoneme restoration play an important role in speech performance in adults with late-acquired deafness. Mental substitution is the ability to integrate the parts of the message that were only partially perceived, and is a skill directly related to cognitive-linguistic development. Therefore, individuals may use mental substitution to complement lip reading (López Álvarez, 2016).

### Cognitive abilities and demographics, habits, medical and psychological factors

Besides hearing loss, other risk factors have been linked to cognitive decline (Livingston et al., 2020).

#### Demographics

##### Age

It is usually considered that above all, cognitive performance is mainly related to age (i.e., the older the patient is, the worse cognition scores) because of the associated physiological neuropathological alterations. The effect of age on cognition has been widely described (Völter et al., 2020; Kang et al., 2021). Here, it comes into play the “common cause hypothesis,” advocating a common factor as the responsible for the continuous decline in physiological structures with aging. According to this hypothesis, common essential mechanisms such as the effects of the aging brain or the age-related cerebrovascular disorder are caused by both deafness and cognitive decline (Amieva and Ouvrard, 2020). At first, this theory affirmed that sensory perception (both hearing and vision) could be a marker of physiological brain integrity (Lindenberger and Baltes, 1994).

Some authors have advised that prevention of cognitive impairment could reduce cognitive decline in individuals with *normal aging* (Kang et al., 2021). However, there is a great variance among individuals. This could be why we found no significant association between cognition and age in this study.

##### Sex

It has been reported that biological sex influences the development of dementia, a fact that can be attributed to the differences in brain structure and function between men and women (Sidenkova and Litvinenko, 2021), which could be related to differences in the way males and females solve cognitive tasks (Jäncke et al., 2020). 65% of deaths from Alzheimer’s disease and other forms of dementia are women (WHO, 2020). Nevertheless, men have a shorter life expectancy than women, and as mentioned previously, age is a risk factor for dementia.

In our study, males outperformed females in the “Visuospatial/constructional” RBANS-H domain, perhaps because of documented sex differences in brain activation pattern in this task (Weiss et al., 2003). This study cohort scored lowest in the “Visuospatial/constructional” domain (Figure 3). This task consists of copying a figure while being aware of line orientation. The fact that women scored lower could be related to level of education; since older women have on average less years of formal education than older men (Livingston et al., 2020) due to fewer opportunities for education for women in the past century. But, according to several meta-analysis (Linn and Petersen, 1985; Voyer et al., 1995; Else-Quest et al., 2010), the sex difference in spatial skills shows a moderate advantage for men, but this difference happens in the absence of a spatial education in the schools. Moreover, it has been reported that with a well-designed training (e.g., video game playing), women could have results similar to men (Hyde, 2016).

Sex differences have been described in previous research (Tamayo et al., 2012; Kestens et al., 2021), with authors encouraging to use sex-stratified analysis when cognitive decline is evaluated (Jockwitz et al., 2021); however, others have not found significant difference between males and females in cognitive tasks (Kessels et al., 2008) which is in accordance with the Gender Similarities Hypothesis (e.g., males and females are quite similar on most psychological variables; Hyde, 2005).

##### Educational level

According to Livingston et al. (2020), low education has, along with hearing loss, the greatest influence on cognitive decline of all modifiable risk factor. There is no clear definition of “low education” (how many years of formal education? full-time or part-time years of education?). Indeed, many authors studying cognition have performed education-matched analysis to avoid this influence in their results (Mertens et al., 2020) or even have chosen participants who at least graduated high school (i.e., 12 years of formal education) to ensure they could perform the cognition tests (Kestens et al., 2021).

In our study, participants with more years of formal education had better cognition scores than those with lower educational attainment. The participants with higher levels of education (35%) were around the 42nd percentile according the RBANS-H evaluation table, whereas the mean of the participants with lower levels of education was in the 5th percentile. It is not unusual that well educated people outperform their lower-educated peers, as cognitive tests often score abilities they are trained in. It is also remarkable that this fact is not necessarily related to the ability to solve everyday problems (Rosselli et al., 2022).

Other studies have also affirmed that higher education levels predict greater performance in cognitive tasks (Claes et al., 2018; Sarant et al., 2020; Völter et al., 2020, 2021).

The incidence of cognitive disorders has diminished in certain countries, mainly because of the changes in education, healthy habits, and lifestyle (Livingston et al., 2020), but it is unclear precisely how education protects against dementia (Livingston et al., 2017). It has been proposed that individuals with higher levels of education have a greater “cognitive reserve,” which could impede the development of dementia (Lisko et al., 2021). Cognitive reserve refers to the ability of the brain to remain functional in the presence of cognitive impairment. This hypothesis proposes that a well-developed and structured brain in subjects with higher education levels would resist better and buffer a possible cognitive impairment (Stern, 2012). Moreover, a link between brain structure and education has also been reported in brain imaging researches, based on the theory that larger brain volumes can better resist the consequences of brain injury (Liu et al., 2012).

#### Habits and medical factors

To explain the link between hearing loss and cognition, it has been suggested that vascular risk factors such as hypertension and diabetes may play a role (Lin and Albert, 2014). This is called “the common cause hypothesis,” in which hearing loss and cognitive decline may result from common etiological elements such as microcirculatory insufficiency (Uchida et al., 2019). So, the cardiovascular and neurocognitive mechanisms do not work separately but are related. This theory is supported by recent advances in neuroimaging monitoring which show that high blood pressure is linked to cerebral atrophy, white matter lesions, and a decrease in brain metabolism (Walker et al., 2017).

Based on the assumption that healthy behaviors can protect against dementia (Livingston et al., 2020; Kang et al., 2021), we asked participants about their frequency of physical activity, smoking and drinking habits, as well as diagnosed health conditions of obesity, diabetes, hypertension, and previous head injury. Drawing causal relationships between these factors is not straightforward, as several risk factors influence others. Physical activity reduces the risk of obesity, diabetes, and cardiovascular diseases (Chieffi et al., 2017). Cardiovascular health is influenced by smoking, obesity, and physical activity, among other factors. It has also been shown that traumatic brain injury is associated with increased risk of dementia, and that this risk is still evident more than 30 years post-injury (Nordström and Nordström, 2018).

According to Norton et al. (2014), around 31% of dementia cases in Europe could be attributed to seven potentially modifiable risk factors; namely diabetes, hypertension, obesity, physical inactivity, smoking, depression, and education. In a cohort study of 9,017 participants from the Swedish Twin Registry (Tomata et al., 2020), only hearing loss and diabetes had a significant relationship with dementia. The incidence of risk factors present in our study cohort ranged from 0% (diabetes) to 27% (physical inactivity).

We found that cognitive performance was influenced by physical inactivity (“Delayed memory” domain) and hypertension status (“Attention” domain and total cognition score). The beneficial influence of physical activity on cognition might be due to increased cerebral blood flow positively affecting neuronal plasticity and memory (Belaya et al., 2021). It has been suggested that both cognitive and physical exercise training may be useful to influence age-related cognitive abilities (Jardim et al., 2021). Regarding hypertension, a study of patients being treated for high blood pressure levels over the course of 10 years suggested that lower blood pressure levels were linked to cognitive protection (Hajjar et al., 2017). This association emphasizes the importance of antihypertensive therapy to reduce cognitive decline (Walker et al., 2017).

#### Psychological factors

Hearing impairment may affect psychosocial health, and can lead to psychological disorders as it affects social relationships (Besser et al., 2018). In our study, both social inhibition and depression correlated negatively with cognitive scores.

A meta-analysis of 62,598 people with a 17-year follow-up revealed an association between depressive episodes and dementia (Prince et al., 2014). Similarly, a recent systematic review of 52 studies concluded that cognitive decline was already observed in the first episode of depression (Varghese et al., 2021). In the same study, it was found that participants who had experienced multiple depressive episodes had worse cognition than those who had single episode.

Other systematic reviews and meta-analyses have demonstrated how social contact exerts a protective effect against dementia (Kelly et al., 2017; Evans et al., 2019).

The “cascade hypothesis” could explained these findings. This theory states that hearing impairment implies cognitive decline mediated by loneliness and social isolation since neural responses might be comprised (Maharani et al., 2019). Because of the experience-dependent neuroplasticity of the brain, the change in response to different stimuli is one of its fundamental characteristics. The “use-it-or-lose-it theory” could be applied in this scenario; i.e., if you do not use your listening and processing abilities, you could lose them. Another mechanism linking hearing loss and cognitive impairment is social inhibition, which is related to unhealthy behaviors (e.g., reduced physical activity or smoking), and these factors have been above described through the “common cause hypothesis” as mediators in cognitive decline (Uchida et al., 2019).

### Limitations

There are some limitations of our study that should be taken into account. Firstly, although diabetes is known to be a risk factor for cognitive decline, no individuals within our study cohort reported this condition. As such, we were unable to assess its significance as a risk factor for cognitive decline within a context of hearing impairment. Secondly, the risk factors related to habits (e.g., smoking, physical inactivity) were self-reported by study participants, so some inaccuracy may have occurred. Moreover, the influence of passive smoking, which could also have an effect on cognitive decline, was not evaluated. Thirdly, another risk factor proposed by Livingston et al. (2020) is exposure to air pollution. As all of our participants reside within the same urban area, we expected minimal variability of this factor within our study population, and thus it was not evaluated. Lastly, the small number of participants, all of whom were recruited from the same center covering a specific Health Area, may also reduce the generalizability of our findings. Despite these limitations, our findings demonstrate how the evaluated risk factors influence cognition in severe to profound hearing-impaired participants.

## Conclusion

In this study, we studied whether cognition is influenced by established risk factors in adults with late-onset deafness. In a cohort of patients scheduled to undergo cochlear implantation, we performed cognitive testing and evaluated the prevalence of risk factors for cognition. We observed that educational level has a significant influence, in that individuals with more years of formal education achieved higher cognition scores. Additionally, sex, physical inactivity, and hypertension may also influence cognitive status. An association was found between some domains of cognition and the psychological traits of social inhibition and depression. Long-term studies with more participants would enable us better understand the effects different risk factors on cognitive status.

## Data availability statement

The raw data supporting the conclusions of this article will be made available by the authors, without undue reservation.

## Ethics statement

The studies involving human participants were reviewed and approved by Ethics Committee of La Paz University Hospital (protocol number: PI-2504). The patients/participants provided their written informed consent to participate in this study.

## Author contributions

MC: cognitive evaluation. MC, IS-C, JG, and LL: data collection, data analysis, and writing. All authors contributed to the article and approved the submitted version.

## Funding

This work was supported by a grant (PI16/00079) from Programa Estatal de Generación de Conocimiento y Fortalecimiento del Sistema Español de I + D + I, ISCiii, Spain.

## Conflict of interest

The authors declare that the research was conducted in the absence of any commercial or financial relationships that could be construed as a potential conflict of interest.

## Publisher’s note

All claims expressed in this article are solely those of the authors and do not necessarily represent those of their affiliated organizations, or those of the publisher, the editors and the reviewers. Any product that may be evaluated in this article, or claim that may be made by its manufacturer, is not guaranteed or endorsed by the publisher.
